# Editorial Comment: Comparison of automated irrigation systems using an in vitro ureteroscopy model

**DOI:** 10.1590/S1677-5538.IBJU.2019.0230.1

**Published:** 2020-02-20

**Authors:** Bruno Marroig

**Affiliations:** 1 Universidade do Estado do Rio de Janeiro Departamento de Cirurgia Geral Rio de JaneiroRJ Brasil Departamento de Cirurgia Geral, Universidade do Estado do Rio de Janeiro - UERJ, Rio de Janeiro, RJ, Brasil

## COMMENT

Several automated irrigation systems have been developed at least along the last three decades. In 1996, Sakhadeo et al. reported a new system of irrigation for ureteroscopy and recommended more widespread use of it by urologists (1). Another automated irrigation system controlling pressure and flow showed consistent reduction in mean ureteroscopy time (32% less with a semi-rigid ureteroscope and 53% less with a flexible instrument), probably due to a wider working space and higher improved visibility, allowing easier progression and manipulation of instruments (2). Some devices control pressure flow output and temperature of saline irrigation.

The problem of elevated intrarenal pressure (IRP) is pyelovenous backflow of fluids, bacteria and/ or endotoxins. The safest irrigation method used during ureteroscopy is by gravity, but it is usually insufficient to overcome liquid resistance in a narrow endoscope, especially if there is any instrument inside the working/irrigation channel. Pressurized irrigation bags routinely used, with or without pump systems, may lead to a wide range of intrarenal pressures, reported from 8.27 to 199.35 cm H2O, depending on the irrigation pressure applied (3). In order to achieve better ureteroscope visualization or allow its progression across ureter, pump flush may lead to increased flow and IRP, which can cause ureteral stone push up. Some devices, such as ureteral access sheath and automated infusion/pressure control devices, may influence intrarenal pressure. In general, IRP remains lower than 30 cm H2O when ureteral access sheath is used because it functions as a escape valve, thereby stabilizing the IRP. Similarly, automated infusion/pressure control devices maintain pre-setting IRP with an automated irrigation/suction pump system (3).

The well conducted study by Fedrigon III and collegues (4) makes a comparison between two automated irrigation systems using an in vitro ureteroscopy model. They first analyzed pressure accuracy. The authors showed that both systems overestimated output pressure, which is not necessarily “bad news”. Although poor accuracy may seem disadvantageous at first sight, at least pressure is overestimated. It would be dangerous for the patient if pressure was underestimated, rendering patients vulnerable to the consequences of elevated IRP. With this piece of information, surgeons can decide if they pre-set higher IRP, if necessary, according to the patient's clinical presentation. Automated systems often need more attention, as advised by Butticè and collegues (5). They call for caution when using the Roboflex Avicenna pump, particularly at high speed settings with resulting high-pressure irrigation during flexible ureteroscopy. This means that even with automated systems, high IRP may occur.

They next investigated flow rate. They found slightly higher flow rate for TFS system, while CRF demonstrated a slightly less variable flow, similar to what would be expected from passive gravity irrigation. The minimal irrigation pressure needed to provide an adequate visualization and good instrumentation should be used to mitigate stone migration. An adequate flow rate is notably important in any urologist understanding, but an important issue not usually considered by surgeons is that adequate flow rates help controlling proper temperature during laser lithotripsy. In 2014 Molina and collegues (6) evaluated the temperature profile of laser litithotripsy in two urinary tract *ex vivo* models in *Ovis aries.* Thermography studies found an important increase in wall temperatures of the urothelium and external ureteral during laser activation. Even in all different testing situations, an important conclusion was that temperature increase was significantly higher with non-irrigation. With irrigation, temperature increase is not sufficient to cause any harm to kidney cells (6). More recently, Butticè et al. published a similar conclusion (7).

Irrigation is needed most of the time during ureteroscopy, but attention should be paid when instrument working channel is occupied with a thicker laser fiber or even with a thinner one, but with a basket in it at the same time (8). The space left for irrigation may lead to a very reduced flow rate and increased intrarenal temperature during laser activation (9). In cases where automated irrigation system is used, activation of laser fiber leads to a rapid increase in temperature, especially in heated saline (10). Even with continuous flow, attention is needed because elevated temperature inside renal cavity causes tissue damage. While experienced surgeons may take advantages of such systems, caution is recommended to those surgeons who are not familiar with them.

## References

[1] 1. Sakhadeo NB, Venkatesh R, Trafford P, Parr NJ. A new system of irrigation for ureteroscopy. Br J Urol. 1996;78:639-40. 10.1046/j.1464-410x.1996.19025.x 8944525

[2] 2. Lechevallier E, Luciani M, Nahon O, Lay F, Coulange C. Transurethral ureterorenolithotripsy using new automated irrigation/suction system controlling pressure and flow compared with standard irrigation: a randomized pilot study. J Endourol. 2003;17:97-101. 10.1089/08927790360587423 12689403

[3] 3. Tokas T, Skolarikos A, Herrmann TRW, Nagele U; Training and Research in Urological Surgery and Technology (T.R.U.ST)-Group. Pressure matters 2: intrarenal pressure ranges during upper-tract endourological procedures. World J Urol. 2019;37:133-42. 10.1007/s00345-018-2379-3 29915944

[4] 4. Fedrigon D III, Alshara L, Monga M. Comparison of automated irrigation systems using an in vitro ureteroscopy model. Int Braz J Urol. 2020;46:390-7. 10.1590/S1677-5538.IBJU.2019.0230 PMC7088507 32167702

[5] 5. Butticè S, Proietti S, Dragos L, Traxer O. Are You Familiar with the Flow of the Roboflex Avicenna Pump? Allow Me to Explain. J Endourol. 2017;31:418-9. 10.1089/end.2016.0377 28052697

[6] 6. Molina WR, Silva IN, Donalisio da Silva R, Gustafson D, Sehrt D, Kim FJ. Influence of saline on temperature profile of laser lithotripsy activation. J Endourol. 2015;29:235-9. 10.1089/end.2014.0305 PMC4313406 25154455

[7] 7. Butticè S, Sener TE, Proietti S, Dragos L, Tefik T, Doizi S, Traxer O. Temperature Changes Inside the Kidney: What Happens During Holmium: Yttrium-Aluminium-Garnet Laser Usage? J Endourol. 2016;30:574-9. 10.1089/end.2015.0747 26837810

[8] 8. Patel N, Monga M. Ureteral access sheaths: a comprehensive comparison of physical and mechanical properties. Int Braz J Urol. 2018;44:524-35. 10.1590/S1677-5538.IBJU.2017.0575 PMC5996793 29493185

[9] 9. Yoo J, Lee SJ, Choe HS, Kim HY, Lee JH, Lee DS. Anterograde irrigation - assisted ureteroscopic lithotripsy in patients with percutaneous nephrostomy. Int Braz J Urol. 2019;45:406-7. 10.1590/S1677-5538.IBJU.2018.0238 PMC6541133 30325601

[10] 10. Bach T, Geavlete B, Herrmann TR, Gross AJ. Working tools in flexible ureterorenoscopy--influence on flow and deflection: what does matter? J Endourol. 2008;22:1639-43. 10.1089/end.2008.0184 18620506

